# Mef2d: a novel transcription factor in type 2 allergic lung inflammation

**DOI:** 10.1038/s41392-024-02022-9

**Published:** 2024-11-07

**Authors:** Laura Surace, Christoph Wilhelm, Christian Bode

**Affiliations:** 1 Institute of Clinical Chemistry and Clinical Pharmacology, University Hospital Bonn, Bonn, 53127 Germany; 2 Department of Anesthesiology and Intensive Care Medicine, University Hospital Bonn, Bonn, 53127 Germany

**Keywords:** Innate immune cells, Adaptive immunity, Molecular medicine

In a recent article published in *Science*, Szeto et al. identified myocyte-specific enhancer factor 2d (Mef2d) as a promoter of type 2 immunity and allergic lung inflammation. Using CRISPR screens and orthogonal recombinase-enabled Boolean gene circuitry, the authors revealed that Mef2d modulates GATA3 expression by suppressing Regnase-1, thereby boosting IL-33 signaling and cytokine production.^1^

Tissue-resident innate lymphoid cells 2 (ILC2s), as well as their adaptive counterparts, T helper (Th) 2 cells, are important mediators of type 2 immunity, orchestrated by the cytokines IL-5, IL-9 and IL-13. Type 2 immunity provides protection against parasites and supports tissue repair after injury.^2^ However, aberrant activation of the type 2 response can lead to allergic pathologies such as asthma, reduced pathogen defense, and fibrosis.^2^ First described as a distinct cell type in 2010, ILC2 has come to the fore as a cell type that controls a wide range of tissue-specific immune functions, including the maintenance of adipose tissue metabolism. Activation of this cell type is achieved in an innate manner by stimulation by the tissue-derived cytokines IL-33, IL-25, or thymic stromal lymphopoietin (TSLP) or by factors such as prostaglandins, leukotrienes, or neuropeptides.^3^ Due to their functional overlap with Th2 cells, there is an ongoing debate about their role once adaptive immunity is established. Resolution of this issue has been hampered by the lack of tools to specifically target ILC2 or Th2 cells. Several genetically modified mouse models are available to specifically target ILC2 in unchallenged animals under steady-state conditions. For instance, using animals expressing the *Cre* recombinase under the control of the endogenous IL-5- (*Red5* mice) or neuromedin U receptor 1 (*Nmur1*) locus (*NMUR1-Cre* mice), which are almost exclusively expressed by ILC2s at steady state, can be used. However, these tools lose specificity in the context of type 2 immune activation, as Th2 cells and eosinophils start expressing both IL-5 and NMUR1.^4^

In order to tackle the differential targeting of ILC2 and Th2, Szeto et al. employed a new strategy. They first used a CRISPR screen to identify regulators of GATA3, the master regulator of type 2 immunity, essential for the development and function of both ILC2 and Th2 cells. They isolated common lymphoid progenitors (CLPs) from transcription factor and cytokine reporter mice (*Id2*^BFP^, *Bcl11b*^tdTomato^, *Gata3*^huCD2^, *Rora*^Teal^*, Rorc*^Katushka^) and cultured them to identify the key factors driving the transition from GATA3-low ILC progenitors to GATA3-high ILC2s. This led to the discovery that the transcription factor Mef2d is a modulator of IL-13 and GATA3 in ILC2s. Mef2d deficiency in lymphocytes resulted in diminished cytokine production from ILC2 and Th2 cells, while Mef2d appeared to be dispensable for type 1/17 immunity.

**Fig. 1:**
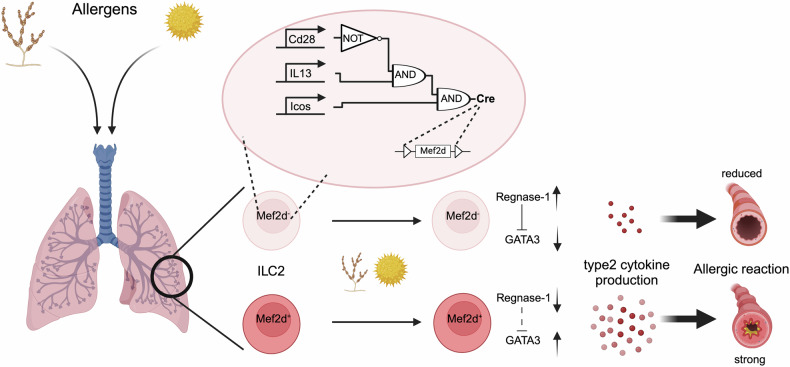
Mef2d promotes allergic type 2 immune responses in the lung. To specifically delete Mef2d in innate lymphoid cells (ILC2), a Boolean strategy was employed by introducing three distinct DNA recombinases into the endogenous loci of ICOS, IL-13, and CD28. In ILC2s, the absence of CD28 expression prevents Vika-mediated recombination at the Il13-Dre locus. As a result, Dre successfully removes the STOP cassette from the Icos-Cre locus, leading to Cre expression and subsequent Cre-mediated modification of the LoxP-flanked locus. In T cells, however, CD28 is expressed, allowing Vika-mediated recombination at the Il13-Dre locus, which deletes the Dre allele flanked by Vox sites. Consequently, Dre cannot remove the STOP cassette from the Icos-Cre locus, resulting in the absence of Cre expression and no Cre-mediated modification of the LoxP-flanked locus. In Mef2d^ILC2KO^ mice, Szeto et al. observed reduced allergic lung inflammation. Mechanistically, Mef2d represses Regnase-1 endonuclease expression, a known negative regulator of GATA3, thereby increasing type 2 cytokine production. Created with BioRender.com and adapted from graphic abstract^1^

To specifically study the role of Mef2d in ILC2 function in vivo, Szeto et al. designed a combinatorial approach for the targeted deletion of Mef2d in ILC2s, allowing them to investigate the function of Mef2d in both innate and adaptive type 2 immune responses (Fig. 1). Three DNA recombinases were introduced into ICOS, IL-13, and CD28 loci. In ILC2s, the lack of CD28 prevents Vika-mediated recombination at the Il13-Dre locus, so Dre removes the STOP cassette from the Icos-Cre locus, leading to Cre expression and modification of the LoxP-flanked locus. In T cells, CD28 is present, allowing Vika-mediated recombination at the Il13-Dre locus, which deletes the Dre allele flanked by Vox sites. Consequently, Dre cannot remove the STOP cassette from the Icos-Cre locus, resulting in no Cre expression or modification of the LoxP-flanked locus.

Challenging *Mef2d*^ILC2KO^ mice with various allergens revealed that Mef2d contributes to type 2 immunity through both ILC2s and Th2 cells, depending on the experimental model used. Deletion of Mef2d from T cells and/or ILCs reduced allergic responses to multiple allergens (Fig. 1). Mef2d deficiency was linked to lower expression of GATA3 and ST2, the IL-33 receptor, in ILC2s. The decreased ST2 expression compromised IL-33 signaling pathways and diminished type 2 cytokine production. This study further revealed that Mef2d acts upstream of GATA3 by repressing the Regnase-1 endonuclease, a negative regulator of both ST2 and GATA (Fig. 1). Since IL-25 activation of ILC2, which also depends on GATA3, is not affected by Mef2d deletion, the impact of Mef2d deletion is context-dependent and reliant on ST2. This is because ST2 is significantly, though not entirely, downregulated by Mef2d deletion. Finally, the authors found that Mef2d associates with the transcription factor NFAT1 and is essential for its effective nuclear translocation and subsequent type 2 cytokine production in response to calcium-dependent signaling.

By using a novel approach, Szeto et al. redefine important aspects of type 2 immunity. Despite CRISPR genome engineering has been extensively used to study adaptive immunity regulation, this study represents the first successful application of high-throughput CRISPR screening to investigate ILC2 function and differentiation. Until now, unbiased screening of rare populations has been challenging due to low cell numbers. By combining two screens, the authors identified both well-known and novel modulators of IL-13 and GATA3 expression during ILC differentiation and development. Mef2d, one of the identified genes, proved to be a key factor contributing to the modulation of GATA3 levels in ILC2. As GATA3 is an essential transcription factor in different immune subsets, not many studies, have focused on defining the nuances of its regulation in adaptive and innate immune cells. By implementing the Boolean logic circuitry to specifically target ILC2 (BIC mice), the authors designed an elegant multi-step approach to temporally and qualitatively dissect type 2 immune responses. Interestingly, while other studies reported a key role of ILC2 in both the initiation and the recall responses to allergens,^5^ Szeto et al. used the BIC mice to prove that ILC2 activity was less important during the recall response. Key questions about immune response dynamics could take advantage of the BIC mouse model system that holds significant potential for studying specific cell types in different disease contexts. Of note, steady-state ILC2 do not express high amounts of IL-13, so the BIC mice as such might not represent the best model to investigate the homeostatic function of ILC2 and the NUMR1-Cre, the KLRG1-Cre, and the Red5 mice, might represent better tools. However, given the flexibility of the BIC logic, a good strategy could be to readapt the BIC mice to target steady-state ILC2 by choosing a different sequence of genes (e.g., IL-5 - ICOS). In the manuscript’s final section, the authors identify the Regnase-Mefd2 axis as a key regulator specifically for lung ILC2, suggesting that tissue-specific programs might define ILC2 biology. Given these findings, it’s crucial to determine the molecular mechanisms behind type 2 immune cell tissue adaptation and to unravel the complex network of pathways influencing their activity in health and disease.
